# Quantitative measures of total and phosphorylated alpha-synuclein in skin tissue as potential biomarkers for synucleinopathies

**DOI:** 10.1177/1877718X261420669

**Published:** 2026-02-26

**Authors:** Bram L van der Gaag, Janna van Wetering, Martino L Morella, Johannes JP Breve, Niels Reijner, Jenna Pfeifer, Amador Simando, JJ van Hilten, Henk W Berendse, Annemieke JM Rozemuller, Marianna Bugiani, Thomas Kustermann, Venissa Machado, Markus Britschgi, Wilma DJ van de Berg

**Affiliations:** 1 Department of Anatomy and Neurosciences, section Clinical Neuroanatomy and Biobanking, Amsterdam UMC, Vrije Universiteit Amsterdam, Amsterdam, The Netherlands; 2 Amsterdam Neuroscience, program Neurodegeneration, Amsterdam, The Netherlands; 3 Delft University of Technology, Delft, The Netherlands; 4 Department of Biology, De La Salle University, Manila, Philippines; 5 St. Luke's Medical Center College of Medicine – William H. Quasha Memorial, Quezon City, Philippines; 6 Department of Neurology, Leiden University Medical Center, Leiden, The Netherlands; 7 Department of Neurology, Amsterdam UMC, Amsterdam, The Netherlands; 8 Department of Pathology, Amsterdam UMC, Amsterdam, The Netherlands; 9 Roche Pharma Research and Early Development, Neuroscience and Rare Diseases Discovery and Translational Area, Biomarker, Roche Innovation Center Basel, F. Hoffmann-La Roche Ltd, Basel, Switzerland; 10 Roche Pharma Research and Early Development, Neuroscience and Rare Diseases Discovery and Translational Area, Research, Roche Innovation Center Basel, F. Hoffmann-La Roche Ltd, Basel, Switzerland

**Keywords:** alpha-synuclein, immunoassay, skin, synucleinopathy, biomarker

## Abstract

**Background:**

Alpha-synuclein can be detected in skin biopsies of individuals with synucleinopathies. However, quantitative data of total and phosphorylated Serine 129 (pS129) alpha-synuclein in skin biopsies are scarce.

**Objective:**

We aimed to investigate the biomarker potential of quantitative total and pS129 alpha-synuclein measurements in skin biopsies from people with synucleinopathies and controls.

**Methods:**

We developed and validated AlphaLISA™ immunoassays to determine total and pS129 alpha-synuclein concentrations. Postmortem skin biopsies of Parkinson's disease (PD: n = 18), Dementia with Lewy bodies (DLB: n = 3), Multiple System Atrophy (MSA: n = 5) and control (n = 5) subjects were collected at the cervical vertebra C7. Brain tissues (middle temporal gyrus and substantia nigra) were collected from these same cases. In addition, skin biopsies of controls (n = 20) and PD cases (n = 40) were obtained from the ProPark cohort.

**Results:**

Total and pSer129 alpha-synuclein could be robustly detected and quantified in all skin samples. We observed a trend towards increased total (+58%, p = 0.055) and pS129 (+131%, p = 0.060) alpha-synuclein skin concentrations in synucleinopathy cases compared to controls. We found no correlations between pS129 alpha-synuclein concentrations in paired brain and skin tissues from the same donors. pS129 alpha-synuclein concentrations were similar for clinical PD cases and controls and there was no correlation with motor symptom severity (UPDRS-III).

**Conclusions:**

These findings highlight that total and pS129 alpha-synuclein can be biochemically quantified in skin biopsies, but warrant further validation and investigation to asses its potential as a diagnostic biomarker in clinical cohorts.

## Plain language summary title

Quantification of alpha-synuclein in skin tissue might provide information that can be used for the detection of Parkinson's disease and related disorders.

**Plain Language Summary:**

In people with Parkinson's disease (PD) or related disorders, together named synucleinopathies, alterations related to the protein alpha-synuclein can often be observed in skin tissue. To what extent this protein differs in skin tissues from people with synucleinopathies compared to people without, has yet to be evaluated. Therefore, we developed an assay to quantify concentrations of alpha-synuclein and a modified form (phosphorylated serine 129) in the skin tissue of people with synucleinopathies and controls after autopsy. Meanwhile, we also looked at alpha-synuclein levels in the brain tissues of these same people. And lastly, we studied alpha-synuclein levels in skin of individuals living with PD and healthy controls. With this assay, we showed that alpha-synuclein forms can be detected and quantified in skin tissue of all investigated cases. The concentration of phosphorylated alpha-synuclein in the skin did not correlate with the concentration observed in the studied brain regions . Skin phosphorylated alpha-synuclein concentrations were similar between subjects living with PD and healthy controls and no correlation was found with disease severity. Overall, this study highlights that alpha-synuclein can be detected and quantified in skin tissues of people suffering from synucleinopathies. More research is needed to determine whether these measures in skin biopsies can be used as a tool for the detection of synucleinopathies in a clinical setting.

## Background

Parkinson's disease (PD) is the fastest-growing neurodegenerative disorder worldwide, with the global prevalence expected to reach 25.2 million by 2050.^1,2^ Neuropathologically, PD is characterized by a progressive loss of dopaminergic neurons in the substantia nigra pars compacta and intracellular abnormal intraneuronal accumulation of the misfolded alpha-synuclein (aSyn) protein into Lewy bodies (LBs) and Lewy neurites (LNs).^3^ Neuronal or glial aSyn inclusions are also present in the brains of people with atypical parkinsonian syndromes, such as Dementia with Lewy bodies (DLB) and Multiple System Atrophy (MSA).^4^ There is a critical unmet need for reliable, accessible, and scalable biomarkers to enable an earlier and more accurate diagnosis of synucleinopathies, to monitor disease progression, and to evaluate therapeutic responses. Recent studies have shown that increased phosphorylated aSyn at serine 129 (pS129 aSyn), a surrogate marker of aggregated aSyn, can be detected not only in cerebrospinal fluid (CSF), but also in peripheral tissues such as the gut, saliva, blood, submandibular glands, kidney and skin.^5–10^ These findings underscore the potential for minimally invasive peripheral tissue-based biomarkers.

A recently proposed biological classification framework includes detection of aggregated aSyn in CSF and skin tissue as confirmatory markers for synucleinopathies.^11^ Among available techniques, immunohistochemistry (IHC) targeting pS129 aSyn in skin biopsies and seed amplification assays (SAAs) for detecting misfolded aSyn seeds in CSF and skin are currently the best validated methods.^11–15^ Regional distribution of skin aSyn pathology seems to be dependent on the type of synucleinopathy, as MSA cases appear to have greater pS129 aSyn deposition and a more widespread peripheral distribution compared to PD cases.^16^ Although SAAs applied to CSF have shown clinical promise, several studies suggest that skin tissue may offer superior diagnostic performance.^17^ In head-to-head comparisons, skin-based SAAs have demonstrated higher sensitivity and specificity for distinguishing synucleinopathies from controls compared to CSF-based assays.^16^ This highlights the viability of skin as a clinically relevant tissue for aSyn biomarker development. Quantitative data of total and pS129 aSyn levels in human skin are however scarce.

To address this, we developed ultrasensitive bead-based AlphaLISA immunoassays for the detection and quantification of total and pS129 aSyn levels and the pS129-to-total aSyn ratio in skin tissue homogenates from indivduals with synucleinopathies and controls. The assays were validated by assessing dilution linearity, inter-assay variability and by determining antibody sensitivity, and specificity. We utilized the novel AlphaLISAs to evaluate total and pS129 aSyn levels in skin biopsies and brain tissue from pathologically confirmed PD, DLB, MSA, and control donors and skin biopsies from individuals with PD and healthy controls from the ‘Profiling Parkinson's’ (ProPARK; www.proparkinson.nl) cohort. We show that total and pS129 aSyn can be detected and quantified in skin biopsies in a reliable and robust way. The differences between groups were not significant, but a trend towards increased total and pS129 aSyn in pathologically-confirmed synucleinopathy cases over controls was observed. In the clinical cohort, we did not detect differences between groups, which illustrates the large variability in quantitative levels of skin pS129 aSyn levels, warranting further investigation in larger cohorts.

## Materials and methods

### Postmortem brain bank cohort

For the current study, 3-mm punch skin biopsies were collected between 2020 and 2024 from brain donors with pathology confirmed PD (n = 18), DLB (n = 3), MSA (n = 5) and non-neurological controls (n = 6). Punches were collected at cervical vertebrae 7 (C7) in a standardized manner. Skin biopsies used for IHC/IF were fixed in buffered formalin (pH 7.4) for 24 h at 4°C after which they were embedded in parrafin. Punches used for biochemical analyses were snapfrozen in liquid nitrogen and stored at −80°C until further use. Postmortem brain tissue from the substantia nigra (SN) and middle temporal gyrus (MTG) from the same cases was obtained via the Netherlands Brain Bank (NBB: http://brainbank.nl) and Normal Aging Brain Collection Amsterdam (NABCA: http://nabca.eu). Donors or the next of kin gave signed informed consent for the donation of skin and brain tissue and the use of medical records for the purpose of the research. The donation programs for the NBB and NABCA were approved by the local ethics committee of the Amsterdam UMC (NBB: 2009.148 and NABCA: 2018.150).

Neuropathological confirmation of the clinical diagnosis was established following the international guidelines of the Brain Net Europe II (BNE) consortium. Clinical and demographic features, including age-at-onset, sex and disease duration were retrieved from the medical records of each case. For the pathological diagnosis, 6-micron thick sections of formalin-fixed paraffin-embedded (FFPE) tissue from the postmortem study cohort were cut and stained using antibodies against aSyn (clone KM51, Monosan Xtra, 1:500 dilution), Aβ (clone 4G8, Biolegend, 1:8000) and phosphorylated tau (clone AT8, ThermoFisher Scientific, 1:500) as previously described.^17^ Thal Aβ phases, Braak neurofibrillary tangle (NFT) stages and Braak aSyn stages were determined.^18–20^ Consortium to Establish a Registry for Alzheimer's Disease (CERAD) neuritic plaques scores were assessed to determine the ABC-scores.^21^ The presence of glial cytoplasmic inclusions (GCIs) was assessed to establish a diagnosis of MSA.^22^ Furthermore, presence of aging-related tau astrogliopathy (ARTAG), primary aging related tauopathy (PART), argyrophilic grains disease (AGD), cerebral amyloid angiopathy (CAA) stages, microvascular lesions and hippocampal sclerosis were assessed.^23,24^ Clinicopathological characteristics and demographics of the cohort are shown in Table 1.

**Table 1.** Clinicopathological information of the postmortem cases.

	Control	PD	DLB	MSA
Number	5	18	3	5
Sex (M/F)	2 / 3	11 / 7	2 / 1	3 / 1
Age at death (years ± SD)	82 ± 7	77 ± 7	78 ± 1	60 ± 7
Disease duration (years ± SD)	n.a.	18 ± 7	8 ± 5	8 ± 6
PMD (hours ± min)	5 ± 110	6 ± 118	7 ± 93	6 ± 58
Thal amyloid phase (range and distribution)	0–3 (3/1/0/1)	0–4 (6/5/5/1/1)	0–3 (1/0/0/2)	0–4 (2/1/0/0/2)
Braak NFT stage (range and distribution)	I-III (3/2)	0-III (5/4/3/6)	II-III (1/2)	0-IV (2/1/0/1/1)
CERAD neuritic score (range and distribution)	0 (5)	0–1 (14/1)^a^	0–3 (1/1/0/1)	0–1 (3/1)^b^
Braak LB stage (range and distribution)	0 (5)	V-VI (1/17)	VI (3)	0-V (2/0/0/0/0/3)

*M* male, *F* female, *SD* standard deviation, *PMD* postmortem delay, *min* minutes, *NFT* neurofibrillary tangle, *CERAD* Consortium to Establish a Registry for Alzheimer's Disease, *LB* Lewy body. ^a^CERAD neuritic score was missing for three PD cases, ^b^CERAD neuritic score was missing for one MSA case.

### ProPark cohort

As part of the multicenter longitudinal ‘Profiling Parkinson's disease’ cohort study (ProPark: https://proparkinson.nl), two skin biopsies were collected at baseline. Ethical approval was given by the local medical ethics committee of the Amsterdam UMC (PI: WvdB, 2019-515). In the current study we included 60 cases, of whom skin biopsies were collected at baseline, from the ProPARK biobank: 40 individuals with PD and 20 age- and sex-matched controls. Clinical measures including Hoehn and Yahr (H&Y) stage, Unified Parkinson's Disease Rating Scale-III (UPDRS-III) and Montreal Cognitive Assessment (MoCA) scores were collected at baseline. Additionally, the baseline levodopa equivalent daily dose (LEDD) was determined for the each PD patient participating in the study.

Three millimeter punch skin biopsies were taken at C7, 5 min after providing local anesthesia (Lidocaine) via subdermal injection by a trained physician. One skin biopsy was fixed in 4% buffered formalin (pH 7.4) for 24 h at 4°C and parrafin embedded for IHC. A second biopsy was frozen immediately after collection using liquid nitrogen and processed for biochemical analysis. Details of the included ProPark cases are provided in Table 2.

**Table 2.** Demographics of the ProPark cohort.

	Control	PD
Number	20	40
Sex (M/F)	10/10	20/20
Age (years ± SD)	68 ± 8	67 ± 7
Disease duration (years ± SD)	n.a.	3 ± 3
H&Y score (mean ± SD)	0 ± 0	2.0 ± 0.4
MoCA score (mean ± SD)	27.9 ± 2.2	27.0 ± 2.4
UPDRSIII score (mean ± SD)	1.9 ± 2.4	24.8 ± 11.6

*M* male, *F* female, *SD* standard deviation, *H&Y* Hoehn and Yahr, *MoCA* Montreal Cognitive Assessment, *UPDRS* Unified Parkinson's Disease Rating Scale, *n.a.* not applicable.

### Immunohistochemistry and immunofluorescence

Pathological aSyn in skin tissue was assessed with IHC on FFPE 20-µm-thick sections. Deparaffinization was performed by sequential incubation in a series of xylene (3 × 10 min) and alcohol (2 × 5 min in 100% EtOH, then 1 × 5 min in 96% EtOH, 80% EtOH and 70% EtOH) and rehydrated for 5 min in demi-water. Endogenous hydrogen peroxidase activity was quenched by incubating the sections with 1% H_2_O_2_ (Merck) for 30 min and non-specific binding sites with 5% Normal Goat Serum (NGS) + 0.5% Triton X-100 for 30 min. Tissue sections were then incubated with primary antibody targeting pS129 aSyn (clone EP1536Y, Abcam, cat# ab51253) at 1:4000 dilution overnight at 4°C in a TBS buffer containing 1% NGS + 0.1% Triton X-100. After overnight incubation, sections were washed 3 × 5 min in TBS after which sections were incubated with an anti-rabbit secondary antibody using the polymer-HRP Envision+™ kit (DAKO, cat#K4003). After secondary antibody incubation, sections were washed 2 × 5 min in TBS and 1 × 5 min in Trish-HCl buffer. DAB was used for chromogen development with H_2_O_2_ and the reaction was stopped after 10 min by washing the sections for 3 × 5 min in Tris-HCl. Heamatoxylin (2 min) was used as counterstaining, following dehydration with 70%, 90%, 100% ethanol and xylene, and coverslipping with Entellan mounting medium (Sigma). For each case, 4 tissue sections were stained from 2 biopsies (2 sections per slide) and 2 tissue sections from 2 biopsies (on 1 slide) was used as a negative control. All investigated sections were adjacent sections, to confirm positivity of pS129 aSyn staining and absence of staining in the negative control section. Skin pS129 aSyn staining was scored blinded and independently by two investigators. Representative images of sections were obtained on the Leica DM5000 microscope using the Leica DFC 450 Camera with either an HC PL APO 40x/1.30 or HC PL APO 63x/1.40-0.60 oil objective.

For immunofluorescence, heat-induced antigen retrieval was performed by steaming sections for 30 min. in 10 mM Tris-EDTA buffer pH 9.0 using a steam cooker (100°C), after deparaffinization. After cooling off the sections to room temperature (RT), sections were washed for 5 min in TBS and incubated with blocking buffer 5% normal goat serum (NGS) in TBS buffer with 0.5% Triton X-100 for 1 h. Next, primary antibody incubation was performed overnight using antibodies against pS129 aSyn (clone 81A, Biolegend, dilution 1:500) and PGP9.5 (clone EPR4118, Abcam, dilution 1:500) at 4°C. After washing 3 × 5 min in TBS and secondary antibody incubation for 1 h with polymer HRP Envision+™ kit and goat anti-rabbit Alexa488 against PGP9.5, DAPI was added as counterstain. Sections were washed 3 × 5 min in Tris-HCl, after which tyramide amplification A647 was performed using H_2_O_2_ as the enzyme catalyst. Hereafter, slides were washed 2 × 5 min in Tris HCl, followed by a final wash for 1 × 10 min in TBS. MOWIOL + DABCO was used as a mounting medium when applying coverslips. The Leica TCS SP8 microscope was used for acquisition, with fluorophores being excited at appropriate wavelengths and the hybrid gating (counting mode) setting being used for detection. Fiji (https://imageJ.nih.gov/ij/) was used for image processing.

### Skin and brain tissue homogenization

Frozen skin tissue biopsies were thawed on ice and washed 3 times using ice-cold 1X PBS to wash away blood. Skin biopsies were then transferred to a cold surface and finely minced using scalpel blades. After mincing, the minced tissue was transferred to a soft tissue CK14 homogenization tube (Bertin Technologies) containing 1.4 mm ceramic beads and homogenization buffer containing 1% Triton X-100, 150 mM NaCl, 5 mM EDTA and phosphatase and protease inhibitors in PBS was added in a 1:20 w:v ratio to the tube. Mechanical homogenization was performed using the Precellys tissue homogenizer with Cryolys cooling attachment for 3 cycles of 30 s at 3800 RPM. After homogenization was performed, samples were rested at 4°C for 5 min and the homogenization procedure, as mentioned above, was performed once more. Homogenates were then transferred to Eppendorf tubes and centrifuged at 10.000 RPM for 3 min. The supernatant was subsequently collected, aliquoted and stored at −80°C until further use for biochemical analyses.

Substantia nigra (SN) and medial temporal gyrus (MTG) frozen tissue was cut into 40 µm sections using a cryostat and collected in a 2 mL Eppendorf tube, and stored at −70°C until further use. For homogenization, tubes were kept on dry ice and then placed in an TissueLyser LT adapter, after which homogenization buffer (1% Triton X-100, 150 mM NaCl, 5 mM EDTA, phosphatase and protease inhibitors in PBS) was added in a 1:20 w:v ratio. A 5 mm steal bead was added to each tube and homogenization was performed at 50 Hz for 2 min. Hereafter, tissue homogenates were centrifuged at 1000 x g for 10 min at 4°C and the supernatant was collected as the crude tissue homogenate, which was aliquoted and stored at −70°C for later use in the biochemical assays.

### AlphaLISA immunoassay for total and phosphorylated Serine 129 alpha-synuclein

In order to quantify total and pS129 aSyn levels, we performed an ultrasensitive bead based immunoassay using the AlphaLISA^®^ platform (PerkinElmer^®^).^25^ Firstly, AlphaLISA acceptor beads (Cat# 6772001, Revvity) were conjugated with either MJFR1 antibody (Cat# ab138501, Abcam) for the measurement of total aSyn or monoclonal antibody asyn-142 (courtesy of Roche) for the measurement of pS129 aSyn, and coupled in a 1:10 coupling ratio.^26^ Beads were first washed with PBS and subsequently centrifuged at 16,000 x g for 15 min after which the supernatant was discarded. Conjugation took place in an Eppendorf tube with the following reagents: 1 mg of AlphaLISA acceptor beads, 0.1 mg of antibody, 1.25 µL of 10% Tween-20, 10 µL of a 20 mg/mL solution of NaBH_3_CN and a final volume of 200 µL was obtained by adding 0.13 M phosphate buffer pH 8.0 to the reaction mixture. Beads were then incubated for 18–24 h at 37°C under slight agitation. Hereafter, blocking was performed by adding 10 µL of freshly prepared carboxy-metholxylamine (CMO) in a 0.8 M NaOH solution and incubation took place for 1 h at 37°C. After blocking, tubes were centrifuged for 15 min at 16,000 x g, after which the supernatant was removed and the pellet was resuspended in 200 µL of 0.1 M Trish-HCl pH 8.0 buffer. This step was repeated another time and, after the last centrifugation step, beads were resuspended at 5 mg/mL in PBS with 0.05% Proclin-300 as a preservative. Beads were kept at 4°C until further use.

Serial dilutions of recombinant proteins were prepared to determine protein concentrations in the skin tissue homogenates. Recombinant full length aSyn protein and pS129 aSyn peptide (courtesy of Roche) were diluted in a 3-fold dilution series ranging from 100 ng/mL to 188 fg/mL in AlphaLISA assay buffer (Revvity, cat#AL000C). For the measurement of pS129 aSyn, skin tissue homogenates were diluted in a 1:5 ratio and brain tissue homogenates (SN and MTG) were diluted in a 1:50 ratio in AlphaLISA assay buffer. Skin samples were diluted in a 1:20 ratio in AlphaLISA buffer for the measurement of total aSyn. For both recombinant standards and samples, 5 µL was loaded per well in a 384-well plate and standards and samples were loaded in triplicate. Hereafter, 10 µL of pre-diluted MJFR1 conjugated acceptor beads for total aSyn or SYN142 conjugated acceptor beads for pS129 aSyn were added to each well at a final concentration of 10 µg/mL. The plate was then sealed, placed on an orbital shaker and shaken for 1 min at RT with 600 RPM. Thereafter, samples were centrifuged using a tabletop plate centrifuge and incubation took place for 2 h at RT. Following incubation, 10 µL of pre-diluted A15115A-biotin antibody (cat#848306, BioLegend) was added to each well at a final concentration of 3 nM. The plate was sealed and shaken as before, after which incubation took place for 1 h at RT. After the final antibody incubation step, 25 µL of pre-diluted streptavidin labeled AlphaLISA donor beads (cat #6760002S, Revvity) was added to each well to a final concentration of 40 µg/mL. The plate was again sealed and shaken as before, after which incubation took place for 30 min at RT. After this final incubation step, the plate was read using the VICTOR^®^ Nivo™ multimode plate reader using the following settings: excitation 660 nm, emission 575/110 nm, excitation time 50 msec, emission time 700 ms, power 100%.

### Data analysis and statistics

Raw data were organized and cleaned in Microsoft Excel. Triplicates were assessed and outliers were removed if CV > 15%. If CV remained >15% after outlier removal, the datapoint was excluded from the analysis. Data were analyzed using Graphpad Prism and aSyn concentrations were extrapolated from raw data values (sigmoidal, 4PL or sigmoidal, 5PL). Normality was assessed by performing Shapiro-Wilk test and examining QQ-plots. Fisher's exact test was used to assess whether male and female ratios were equal between groups. Group comparisons were performed by doing a one-way ANOVA with Tukey's multiple comparisons test to compare control, DLB, MSA and PD groups or Kruskall-Wallis with Dunn's correction for multiple comparisons in case of non-Gaussian distribution of the data. MSA, DLB and PD were also pooled as ‘synucleinopathies’ and compared to controls by performing an unpaired t-test if data followed a Gaussian distribution. The Mann-Witney test was performed as a non-parametric alternative. Receiver operating characteristic (ROC) analyses were conducted to evaluate the diagnostic performance of the assay. The area under the curve (AUC) was calculated to determine the sensitivity and specificity for distinguishing patient groups (MSA, DLB, en PD) from controls as well as for differentiating individuals with synucleinopathies as a single group from controls. Spearman's correlation analysis was performed to asses whether two different outcome measures showed any correlation to each other and data was visualized by plotting a linear regression line with 95% confidence interval bands. Significant differences were considered with p < 0.05. Considering the limited size of our cohorts and the explorative nature of this study, trends were reported if 0.05 < p < 0.10.

## Results

### Demographics of the included brain donors and ProPARK subjects

We included postmortem skin and brain tissue, collected at autopsy, from donors with a synucleinopathy, including 18 pathologically confirmed PD cases, 3 DLB cases, 5 MSA cases and 5 controls. Demographics are summarized in Table 1. Group comparisons revealed that MSA cases were significantly younger at death compared to controls (p < 0.0001), PD (p = 0.0001) and DLB (p = 0.003) cases. Disease duration was significantly longer in PD versus MSA cases (p = 0.036), but not compared to DLB. CERAD neuritic plaque scores were significantly higher in DLB cases versus PD (p = 0.037) cases. Braak aSyn stages were significantly higher in PD cases than in controls (p < 0.0001) and MSA cases (p = 0.003). Additionally, Braak aSyn stages were significantly higher in DLB cases compared to controls (p = 0.011). Full details on the clinicopathological characteristics of the postmortem cohort can be found in supplementary table 1.

PD patients had received a clinical diagnosis according to established MDS clinical diagnostic criteria, evaluated by a movement disorders specialist (for demographics see Table 2).^27^ UPDRS III scores (p < 0.0001) were significantly higher in PD cases compared to controls but MoCA scores were similar in PD and control cases (p > 0.05.). All PD patients received L-DOPA medication. Full clinical details, including H&Y scores and LEDD, of the ProPark cohort at baseline can be found in supplementary table 2.

### pS129 aSyn features in skin tissues from control and synucleinopathy cases

**Figure 1.**
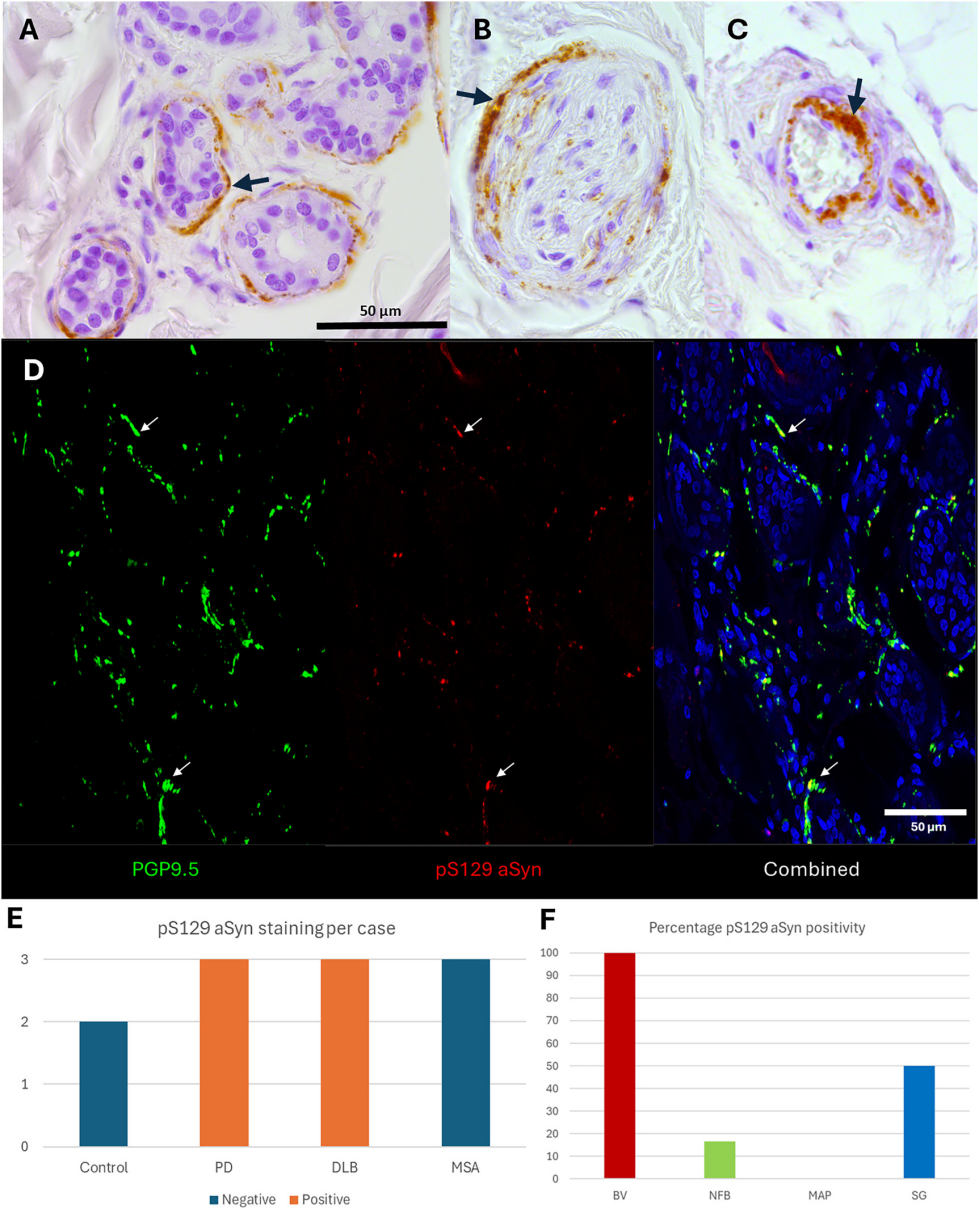
Immunohistochemistry and immunofluorescence stainings for pS129 aSyn on postmortem human skin sections. Representative images of immunohistochemistry for pS129 aSyn highlighting deposits (arrows) in sweat glands (A), nerve fiber bundles (B) and blood vessels (C) visualized with DAB staining. Immunofluorescence staining highlights the presence of pS129 aSyn deposits in PGP9.5^+^ nerve fiber bundles (arrows) in sweat glands, while DAPI was used to visualize cell nuclei (D). Scoring skin pS129 aSyn staining (DAB) in pathologically confirmed cases, we found 3/3 PD and DLB cases to be positive, while MSA (0/3) and control 0/2 cases showed no staining in the investigated sections (E). Evaluating which dermal structures showed pS129 aSyn positivity in cases with pS129 aSyn positive features, we identified blood vessels (BV) to have the highest positivity rate (100%), followed by sweat glands (SG 50%) and nerve fiber bundles (NFB; 16.6%), while no staining was identified in muscle arrector pili (MAP; 0%) structures (F). Scale bars in both the immunohistochemistry and immunofluorescent images represent 50 µm.

By IHC staining, we investigated pS129 aSyn features in the skin of a subset of synucleinopathy cases (n = 3 for PD, DLB and MSA each) and controls (n = 2). We identified pS129 aSyn staining patterns in sweat glands (SG; Figure 1A), nerve fiber bundles (NFB; Figure 1B) and blood vessels (BV; Figure 1C) but not in muscle arrector pili (MAP). By IF, we confirmed that pS129 aSyn was located in PGP^+^ nerve fibers innervating these structures (Figure 1D). All PD (3/3 cases) and DLB (3/3 cases) showed immunoreactivity in FFPE skin tissue sections for pS129 aSyn, while MSA (0/3) and control (0/2) cases did not reveal any pS129 features (Figure 1E). Interestingly, while we did not observe pS129 aSyn pathological features in the skin of any of the MSA cases, 2/3 cases had a Braak aSyn stage of >4 (Figure 1E). Determining the localization of skin pS129 aSyn staining in positive cases, we found that 100% of cases cases showed staining in the BV (Figure 1F). For SG, pS129 aSyn was observed in 50% cases (Figure 1F). NFB showed showed pS129 aSyn staining in 16.6% of cases (Figure 1F). Lastly, MAP revealed no pS129 aSyn staining in any of the investigated sections (Figure 1F).

### Validation of total and pS129 aSyn AlphaLISA immunoassays in skin tissue homogenates

As previously shown, MJFR1 total aSyn acceptor beads were able to detect 76% of recombinant pS129 aSyn and 100% recombinant unmodified monomeric aSyn, while the syn142 pS129 aSyn acceptor beads detected 100% of recombinant pS129 aSyn and 0% of recombinant unmodified monomeric aSyn.^25,26^ After establishing the calibration curve, the lower limit of detection (LLOD) for total aSyn was determined to be 7.86 pg/mL and the lower limit of quantification (LLOQ) was determined to be 13.8 pg/ml (supplementary figure S1A). Inter-assay variability for total aSyn measurements was assessed for n = 5 samples, and was deemed acceptable between runs, with a mean coefficient of variant (CV) = 14.5%, although 3/5 cases were above CV > 15% (supplementary figure S1B). Dilution linearity for total aSyn was confirmed in skin samples at 1:10 (set at 100% recovery), 1:30 (mean recovery = 131%) and 1:90 (mean recovery = 114%) dilutions (supplementary figure S1C). For pS129 aSyn, the LLOD was determined to be 1.06 pg/mL and the LLOQ was determined to be 3.05 pg/mL (supplementary figure S1D). Inter-assay variability for pS129 aSyn measurements was acceptable between runs with a mean CV = 6.93% (supplementary figure S1E). Dilution linearity for pS129 aSyn was confirmed in skin samples with high analyte concentrations at dilutions 1:5 (set at 100% recovery) and 1:10 (mean recovery = 93%), but recovery was suboptimal at a dilution of 1:20 (mean recovery = 68.3%), due to concentrations of the samples being near the LLOQ (supplementary figure S1F).

### Total and pS129 aSyn levels in postmortem skin tissue homogenates of synucleinopathy and control cases

**Figure 2.**
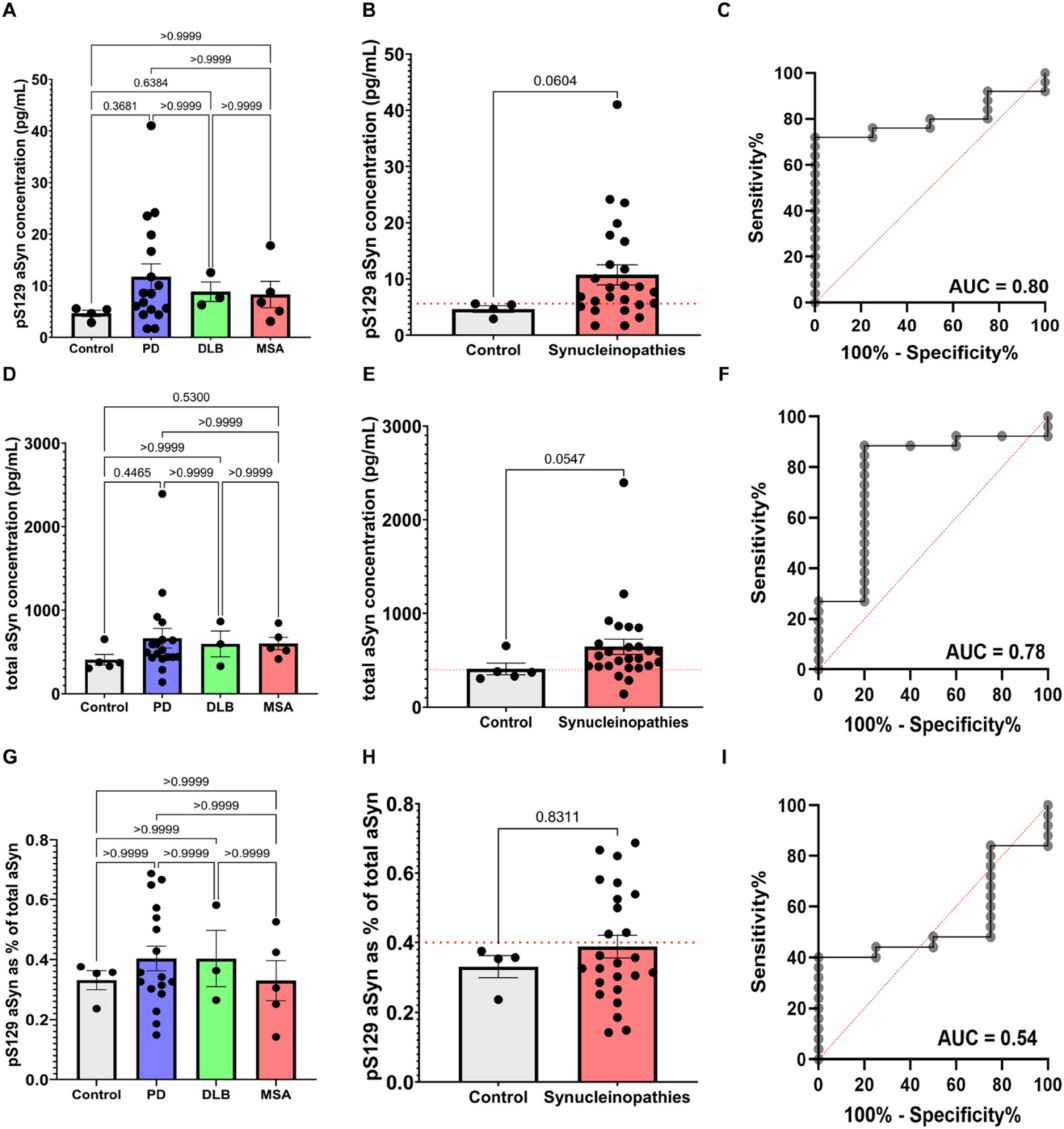
Quantitative skin pS129 and total aSyn measurements in postmortem cases. Skin pS129 aSyn measurements revealed no significant differences between any of the groups (A) but a trend was observed towards elevated concentrations in pooled synucleinopathies versus the control cases (B). ROC curve analysis revealed an AUC of 0.80 for distinguishing synucleinopathies from controls using skin pS129 aSyn concentration (C). Skin total aSyn concentrations were similar between all groups (D) but a trend was observed for increased concentrations when comparing pooled synucleinopathies versus controls (E). ROC curve analysis revealed an AUC of 0.78 for distinguishing between synucleinopathies and controls using skin total aSyn levels (F). Skin pS129/total aSyn ratios (in %) were also similar between groups (G) and there were no differences between pooled synucleinopathies and controls (H). ROC curve analysis revealed an AUC of 0.54 for distinguishing synucleinopathies from controls using skin pS129/total aSyn ratio (I). Graphs show mean value ± standard deviation and red dotted lines display cutoff values used for the ROC curve analyses. AUC = area under curve.

For postmortem total aSyn measurements in skin tissues, mean intra-assay variability was CV = 2.54% and total aSyn concentrations could be determined in 31/31 cases. We observed no differences between PD (n = 18, mean concentration = 665 pg/mL), DLB (n = 3, mean concentration = 597 pg/mL), MSA (n = 5, mean concentration = 601 pg/mL) and control (n = 5, mean concentration = 408 pg/mL) cases (Figure 2A). When comparing the synucleinopathy cases combined (n = 26, mean concentration = 645 pg/mL), we observed a trend towards increased concentrations (+58%, p = 0.055) compared to the control group (Figure 2B). ROC curve analysis revealed a sensitivity of 88% and a specificity of 80% (at >397.8 pg/mL cutoff) with an AUC = 0.78 for distinguishing synucleinopathies from controls using the total aSyn levels (Figure 2C).

For postmortem skin pS129 aSyn measurements, mean intra-assay variability was CV = 4.98% and pS129 aSyn concentrations could be measured in 29/31 cases (1 PD and 1 control case were excluded due to CV > 15%). Similarly, we observed no significant differences in pS129 aSyn concentrations comparing PD (n = 17, mean concentration = 11.8 pg/mL), DLB (n = 3, mean concentration = 8.85 pg/mL), MSA (n = 5, mean concentration = 8.31 pg/mL) and control (n = 4, mean concentration = 4.63 pg/mL) cases (Figure 2D). When all synucleinopathy cases were pooled, pS129 aSyn levels showed a trend towards increased pS129 aSyn concentrations (+131%, mean concentration = 10.7 pg/mL; p = 0.060) compared to controls (Figure 2E). ROC curve analysis revealed a sensitivity of 72% and a specificity of 100% for distinguishing synucleinopathies from controls (at >5.614 pg/mL cutoff, see Figure 2F) with an AUC of 0.80.

Additionally, we examined whether the ratio between pS129/total aSyn would result in a more accurate discrimination between synculeinopathies and control cases. We observed no significant differences between the PD (n = 17, mean pS129 aSyn as % of total aSyn = 0.40%), DLB (n = 3, mean pS129 aSyn as % of total aSyn = 0.40%) and MSA (n = 5, mean pS129 aSyn as % of total aSyn = 0.33%) and control (n = 4, mean pS129 aSyn as % of total aSyn = 0.33%) cases (Figure 2G). In this analysis, we found no differences between the pooled synucleinopathy cases (n = 25, mean pS129 as % of total aSyn = 0.39%) and the control cases (see Figure 2H). ROC curve analysis revealed a sensitivity of 40% and specificity of 100% for distinguishing synucleinopathies from controls (at >0.4% pS129 as a percentage of total aSyn cutoff) with an AUC of 0.54 (see Figure 2I).

**Figure 3.**
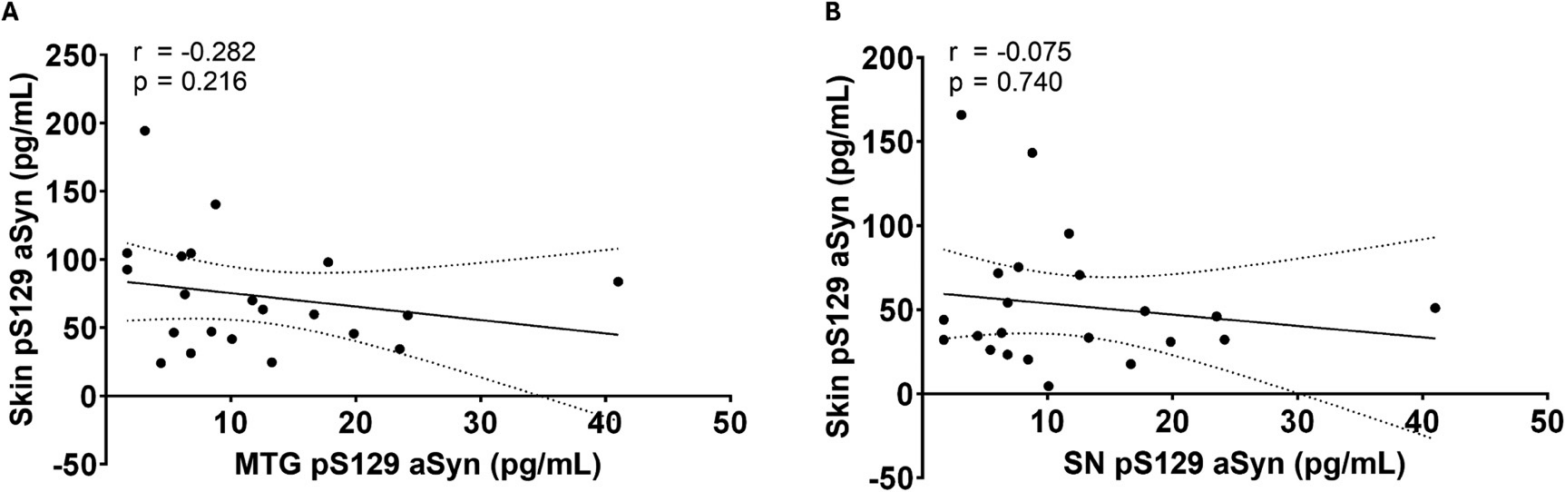
Correlational analyses between brain and skin pS129 aSyn concentrations in postmortem synucleinopathy cases. Spearman's analysis revealed no significant correlation between pS129 aSyn concentrations in the middle temporal gyrus (MTG) and skin tissue of postmortem synucleinopathy cases (A). Similarly, Spearman's analysis also revealed no significant correlation between pS129 aSyn concentrations in the substantia nigra (SN) and skin tissue of these cases (B). Graphs display linear regression lines with 95% confidence interval bands. r = Spearman's rho.

Spearman correlation analysis, revealed no significant correlations between pS129 aSyn levels in the skin and pS129 aSyn levels in the middle temporal gyrus tissue homogenates (Spearman's rho = −0.281, p = 0.216) in the postmortem synucleinopathy subjects (n = 21; see Figure 3A). Similarly, there was no correlation (Spearman's rho = 0.168, p = 0.413) between pS129 aSyn levels in the skin and pS129 aSyn levels in the substantia nigra in postmortem synucleinopathy subjects (n = 22; Figure 3B).

### pS129 aSyn levels in skin tissue homogenates from ProPark cases

**Figure 4.**
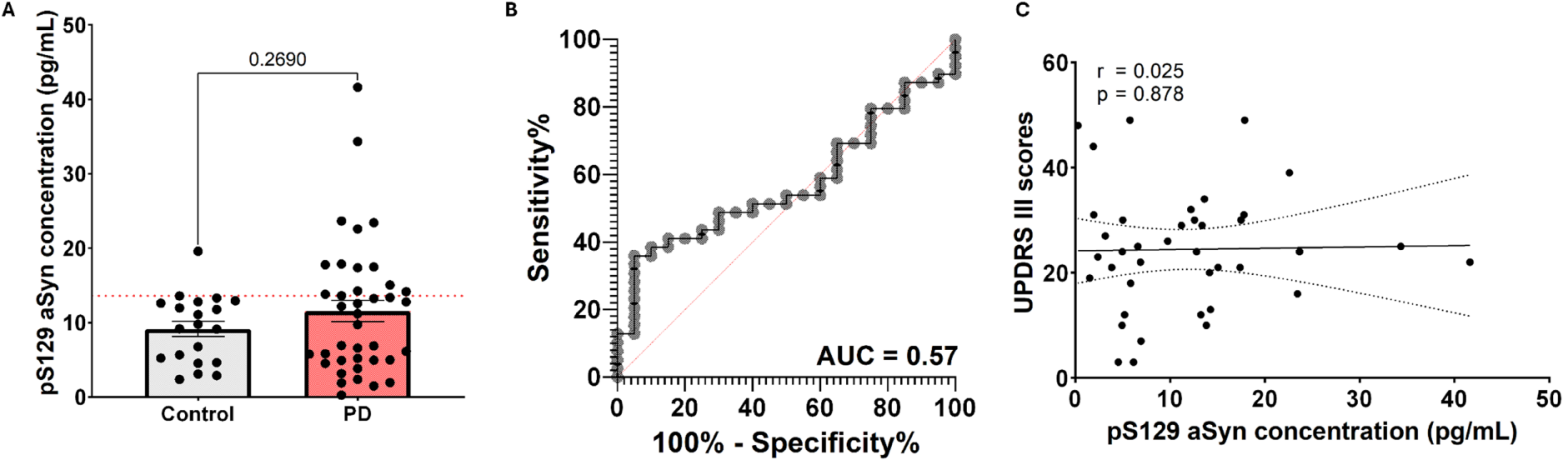
Quantitative skin pS129 aSyn measurements in clinical cohort. Skin pS129 aSyn concentrations did not significantly differ between PD and control cases in the ProPark cohort (A). ROC curve analysis using skin pS129 aSyn concentrations revealed an AUC = 0.57 for distinguishing synucleinopathies from controls (B). Skin pS129 aSyn concentrations revealed no significant correlation with motor symptom severity (UPDRS III scores) in the clinical PD cases. Graph A shows mean value ± standard deviation and the red dotted line displays cutoff value used for the ROC curve analyses. Graph C displays linear regression line with 95% confidence interval bands. AUC = area under curve, r = Spearman's rho.

Since postmortem pS129 aSyn levels in skin indicated potential differences between synucleinopathy and control subjects, we selected pS129 aSyn levels as a measure to be evaluated in the ProPark skin tissue samples. Mean intra-assay variability was CV = 5.85% and pS129 aSyn concentrations were measured in 59/60 cases (1 PD case was omitted from the study design). The analysis revealed similar average pS129 aSyn concentrations in PD (n = 39, mean concentration = 11.5 pg/mL) and control (n = 20, mean concentration = 9.1 pg/mL) cases (see Figure 4A). ROC curve analysis revealed a sensitivity of 36% and specificity of 95% for distinguishing PD cases from controls based on skin pS129 aSyn levels (>13.6 pg/mL cutoff) with an AUC = 0.57 (see Figure 4B). Noteworthy, we found no significant correlation (Spearman's rho = 0.025, p = 0.878) between pS129 aSyn levels and UPDRSIII scores in clinical PD cases (n = 39; Figure 4C). Additionally, we found no correlations between skin pS129 aSyn concentrations and H&Y scores, MoCA scores and LEDD (p > 0.05).

## Discussion

In the current study, we highlight the potential of quantitative detection of total and pS129 aSyn in cervical skin tissue using novel in-house developed AlphaLISAs. We showed that total and pS129 aSyn concentrations are detectable in skin biopsies with high analytical sensitivity and excellent reproducibility (inter-assay variability < 15% CV). In our postmortem cohort, we found trends towards increased total and pS129 aSyn levels in postmortem skin biopsies from synucleinopathy cases compared to controls and no correlation between skin and brain samples. pS129 aSyn levels in PD patients from the ProPark cohort did not differ significantly from controls and pS129 aSyn levels did not correlate with motor symptom severity (UPDRS III scores). Our in-house developed and validated quantitative aSyn and pS129 aSyn AlphaLISAs can be utilized in follow-up studies to investigate the biomarker potential of quantitative cutaneous levels of total and pS129 aSyn in larger cohorts and at different disease stages.

The results of the biochemical evaluation of pS129 aSyn in skin tissue of synucleinopathy cases are twofold: on the one hand, we found an AUC = 0.80 for distinguishing synucleinopathies from controls in postmortem skin biopsies, but only an AUC = 0.57 for distinguishing PD from controls in the ProPark cohort. This difference could be explained by variety of reasons: 1) postmortem skin samples contain less blood compared to *in vivo* samples and since erythrocytes are a major source of aSyn, this could have influenced our findings; and 2) pS129 aSyn features in the skin are more likely present in end-stage than early-stage disease for some cases, as recent evidence suggest that aSyn aggregation could either start in the peripheral/enteric nervous system (body-first) and spread to the central nervous system or vice versa (brain-first).^8,28,29^ However, it should be noted that an earlier study found both an increase in pS129 aSyn in both the cytosolic and membrane fraction of erythrocytes of PD cases compared to controls, making the former explanation less likely to be the case.^8^ A recent study investigating pS129 aSyn immunopositivity in skin tissues (C7) indeed found a significantly higher rate of immunopositivity in body-first (92.4%) versus brain-first (61.9%) cases.^30^ In the clinical cohort, there were a few cases which showed markedly higher concentrations compared to the mean of the control group, which potentially indicates that these cases could represent body-first cases. Interestingly, a recent study investigating pS129 aSyn cutaneous deposition in patients with idiopathic REM sleep behaviour disorder (iRBD), found deposits in 75% of iRBD cases, whereas no deposits were found in the control group, inidicating that alterations in cutaneous pS129 aSyn expression can be detected in a prodromal body-first stage of PD.^31,32^ Changes to the homogenization procedure by using different types of buffer and ultracentrifugation to separate soluble from insoluble proteins, might improve the separation between PD and control cases in the clinical cohort.^17,26^

Comparing these results to earlier studies in which pS129 aSyn was analyzed in clinical skin tissue, we found some interesting differences. In a recent study investigating pS129 aSyn in clinical cases by IF, 54/55 PD, 48/50 MSA and 48/50 DLB showed pS129 aSyn positivity, while only 4/120 controls were immunopositive.^13^ This differs from our IHC results, where we did find pS129 aSyn deposits in PD and DLB cases but not in MSA and controls. It should be noted in the study by Gibbons and colleagues, a much larger tissue volume was examined (6 tissue sections of 50 µm thickness per skin biopsy) using confocal microscopy.^13^ More in alignment with our results, another study using IHC to analyze pS129 aSyn deposition in skin tissues from PD, MSA and control subjects, found pS129 aSyn immunopositivity in 10/10 PD cases but 0/10 for MSA and control cases.^33^ Considering these conflicting findings, a recent systematic review with meta-analysis highlighted that using skin alpha-synuclein IHC and IF resulted in poor pooled specificity to distinguish PD from MSA (specificity = 0.23 and 0.28 respectively), with immunology studies showing a range in specificity from 0.00 to 1.00.^15^ A more appropriate approach to distinguish PD from MSA using skin tissue might be to assess pS129 aSyn staining in Remak non-myelinating Schwann cells, as these cells seem to be exclusively affected in MSA and not in PD/DLB.^34^ Moreover, it seems that somatic nerves in particular are affected by pS129 aSyn deposition in MSA, which we did not assess in the current study, while in PD/DLB cases mainly autonomic nerves are affected^34–36^ Discrepancies between studies with respect to sensitivity and specificity for detecting pS129 aSyn in synucleinopathy cases by IHC/IF could be due to differences in thickness of used tissue sections, processing procedures (type of fixative used, duration of fixation, use of frozen vs parrafin embedded tissue) and antibody selection.^37–39^

We hypothesized that pS129 aSyn levels in the skin would correlate with pS129 levels in the brain of the same donor, serving as a window into aSyn brain pathology.^40^ However, we found that pS129 skin aSyn concentrations did not correlate with pS129 aSyn concentrations in the brain tissue homogenates at autopsy. Neither did we find a correlation between pS129 aSyn skin levels and severity of motor symptoms in the clinical samples. These results suggest that skin pathology may not be suitable as a proxy for the severity of brain pathology in patients. Interestingly, a recent study investigating the effects of a phospholipid curcumin formulation showed reduced skin pS129 aSyn load in the treated PD group versus the control group, suggesting that skin levels may reflect disease state.^41^ Meanwhile, the SAA has the main limitation of being a qualitative assay, limiting its use to track disease progression, although recent efforts show that is is possible to quantify aSyn aggregates after seeding in CSF and brain samples using antibodies.^42,43^ Larger clinical studies in well-characterized cohorts are needed to further evaluate the potential of pS129 aSyn skin concentrations as biomarker for early diagnostics or evaluation of disease state.

Strengths of the current study include the use of standardized protocols for collection of the skin biopsies, pathologically confirmed postmortem brain tissue and skin biopsies from the same donor, allowing for a direct comparison between aSyn values in skin versus brain. Moreover, we developed and validated an ultrasensitive immunoassay in-house using the AlphaLISA™ platform and showed reliable detection and quantification of the analytes in our skin and brain samples. Limitations include the size of the cohorts, as for both the postmortem and clinical cohort the number of cases that were included was limited, making statistical analyses difficult and extrapolation of the findings limited. A natural limitation of working with clinical cases is that there is no pathological confirmation of the clinical diagnosis and the lack of certainty that the control cases do not harbor any aSyn pathology.^44,45^ Additionally, we have not performed a direct comparison with a skin aSyn SAA, as quantitative SAA assays are still under development. This would have been of value to allow an assessment of the validity of our newly developed method over more established techniques within the aSyn biomarker field. Such a comparison will have to await a study with a larger sample size, in order to have the necessary statistical power.

## Conclusions

In the current study, we highlight the development and validation of novel ultrasensitive immunoassays for the detection and quantification of total and pS129 aSyn in human skin tissues. We highlight that biochemical quantification of pS129 aSyn in skin tissue holds potential for distinguishing synucleinopathies from controls. Larger studies with deep phenotyping and standardized protocols for collection of skin biopsies are needed to study the biomarker potential of quantitative levels of skin pS129 aSyn.

## Supplemental Material

sj-docx-1-pkn-10.1177_1877718X261420669 - Supplemental material for Quantitative measures of total and phosphorylated alpha-synuclein in skin tissue as potential biomarkers for synucleinopathies Supplemental material, sj-docx-1-pkn-10.1177_1877718X261420669 for Quantitative measures of total and phosphorylated alpha-synuclein in skin tissue as potential biomarkers for synucleinopathies by Bram L van der Gaag, Janna van Wetering, Martino L Morella, Johannes JP Breve, Niels Reijner, Jenna Pfeifer, Amador Simando III, JJ van Hilten, Henk W Berendse, Annemieke JM Rozemuller, Marianna Bugiani, Thomas Kustermann, Venissa Machado, Markus Britschgi and Wilma DJ van de Berg in Journal of Parkinson's Disease

## Abbreviations

aSyn: alpha-synuclein
pS129: phosphorylated Serine 129
PD: Parkinson's disease
MSA: multiple system atrophy
DLB: dementia with Lewy bodies
SN: substantia nigra
MTG: middle temporal gyrus
LLOD: lower limit of detection
LLOQ: lower limit of quantification
LB: Lewy body
LN: Lewy neurite
CSF: cerebrospinal fluid
IHC: immunohistochemistry
IF: immunofluorescence
SAA: seed amplification assay
C7: cervical vertebra 7
ROC: receiver operating characteristic
UPDRS: Unified Parkinson's Disease Rating Scale
MDS: movement disorder society
FFPE: formalin fixed and paraffin embedded
AUC: area under curve
iLBD: incidental Lewy body disease
iRBD: idiopathic REM sleep behavior disorder
SG: sweat gland
NFB: nerve fiber bundle
MAP: muscle arrector pili
BV: blood vessel
