# Supplementary material for: Quantitative measures of total and phosphorylated alpha-synuclein in skin tissue as potential biomarkers for synucleinopathies
Source: J Parkinsons Dis. 2026 Feb 26;16(3):438–51. doi: 10.1177/1877718X261420669 (PMC13347601; doi:10.1177/1877718X261420669)
Supplement: sj-docx-1-pkn-10.1177_1877718X261420669 - Supplemental material for Quantitative measures of total and phosphorylated alpha-synuclein in skin tissue as potential biomarkers for synucleinopathies [file sj-docx-1-pkn-10.1177_1877718X261420669.docx]

**Supplementary file**

Quantitative measures of total and phosphorylated alpha-synuclein in skin tissue as potential biomarkers for synucleinopathies

*Bram L. van der Gaag^1,2^, Janna van Wetering^1,2^, Martino L. Morella^1,2^, Johannes J.P. Breve^1,2^, Niels Reijner^1,2^, Jenna Pfeifer^1,3^, Amador Simando III,^1,4,5^, J.J. van Hilten^6^, Henk W. Berendse^2,7^, Annemieke J.M. Rozemuller^2,8^, Marianna Bugiani^2,8^, Thomas Kustermann^9^, Venissa Machado^9^ , Markus Britschgi^10^, Wilma D.J. van de Berg^1,2^*

^1^Amsterdam UMC, Vrije Universiteit Amsterdam, department of Anatomy and Neurosciences, section Clinical Neuroanatomy and Biobanking, Amsterdam, The Netherlands

^2^Amsterdam Neuroscience, program Neurodegeneration, Amsterdam, The Netherlands

^3^Delft University of Technology, Delft, The Netherlands

^4^De La Salle University, Department of Biology, Manila, Philippines

^5^St. Luke’s Medical Center College of Medicine – William H. Quasha Memorial, Quezon City, Philippines

^6^Leiden University Medical Center, department of Neurology, Leiden, The Netherlands

^7^Amsterdam UMC, department of Neurology, Amsterdam, The Netherlands

^8^Amsterdam UMC, department of Pathology, Amsterdam, The Netherlands

^9^Roche Pharma Research and Early Development, Neuroscience and Rare Diseases Discovery and Translational Area, Biomarker, Roche Innovation Center Basel, , F. Hoffmann-La Roche Ltd., Basel, Switzerland

^10^Roche Pharma Research and Early Development, Neuroscience and Rare Diseases Discovery and Translational Area, Research, Roche Innovation Center Basel, F. Hoffmann-La Roche Ltd., Basel, Switzerland

Corresponding author:

Name: Bram L. van der Gaag

E-mail: b.l.vandergaag@amsterdamumc.nl

Telephone: +31681733624

ORCID: https://orcid.org/0000-0002-9880-6525

**Supplementary table 1. Clinicopathological information of postmortem donors.** PMD = postmortem delay, NFT = neurofibrillary tangle, LB = Lewy body, CAA = cerebral amyloid angiopathy, ARTAG = aging related tau astrogliopathy, PART = primary aging related tauopathy, AGD = agyrophilic grain disease, n.ap. = not applicable, n.av. = not available, - = missing.

**Supplementary table 2. Clinical information of ProPARK donors at baseline.** Age, age at onset and disease duration is depicted in years. H&Y = Hoehn and Yahr, UPDRS = Unified Parkinson’s Disease Rating Scale, MoCA = Montreal Cognitive Assessment, LEDD = Levodopa Equivalent Daily Dose, n.ap. = not applicable, n.av. = not available.

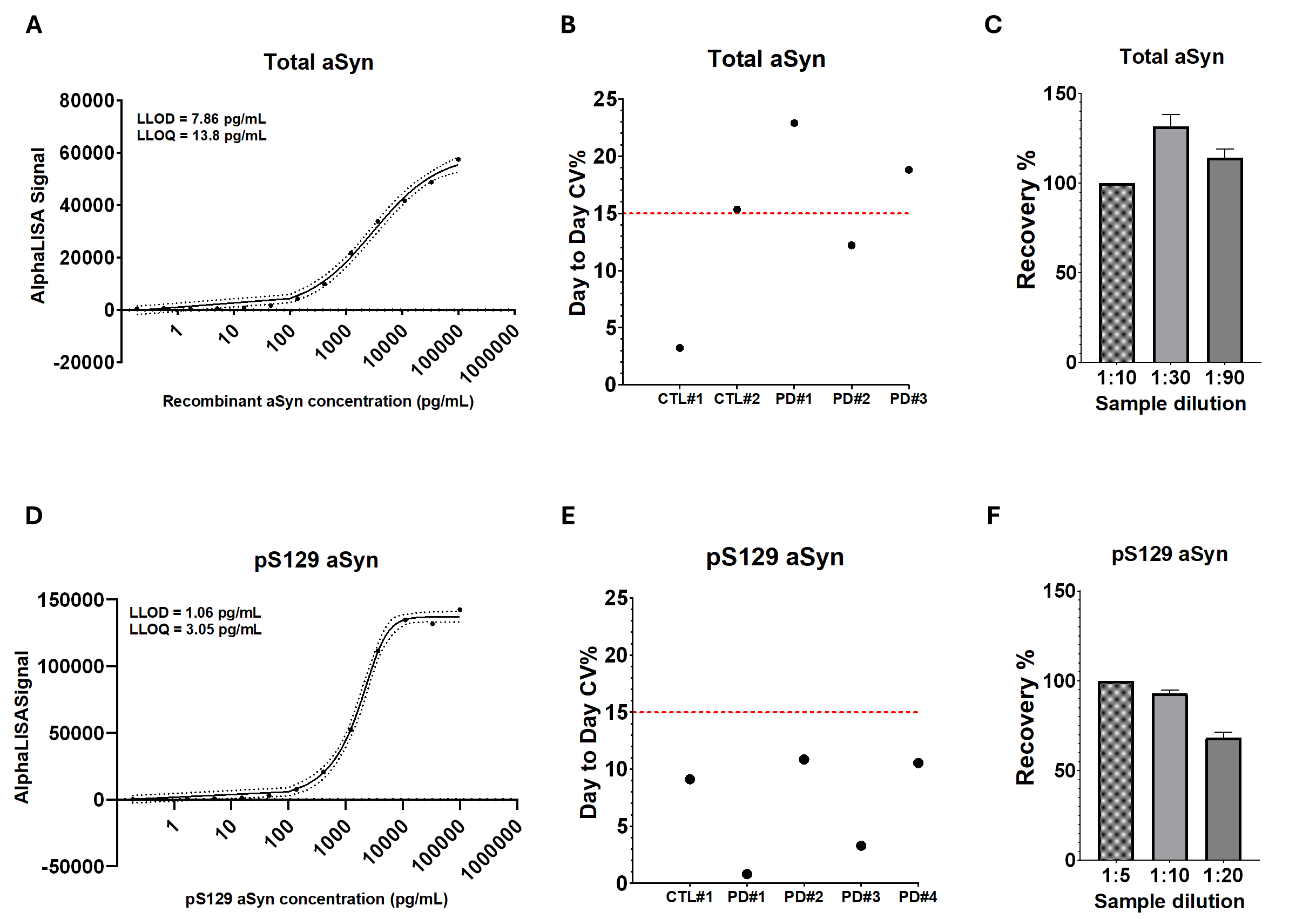


**Supplementary figure S1. Total and pS129 aSyn AlphaLISA assay development and validation for skin tissue homogenates.** Lower limit of detection (LLOD) was determined to be 7.86 pg/mL and 13.8 pg/mL was determined to be the lower limit of quantification (LLOQ) for measuring total aSyn (A). Mean inter-assay coefficient of variation (CV) was 14.5% for total aSyn measurements in skin tissue samples across experiments (B). Dilution linearity was assessed for total aSyn at 1:10 (set at 100% recovery), 1:30 (mean recovery = 131%) and 1:90 (mean recovery = 114%) dilutions of skin tissue homogenates in AlphaLISA assay buffer (C). For pS129 aSyn, LLOD was determined to be 1.06 pg/mL and 3.05 pg/mL for the LLOQ (D). Mean inter-assay CV was 6.93% for pS129 aSyn measurements in skin tissue samples across experiments (E). Dilution linearity was assessed for pS129 aSyn measurements at 1:5 (set at 100% recovery), 1:10 (mean recovery = 93%) and 1:20 (mean recovery = 68%) dilutions of skin tissue homogenates (F). AlphaLISA calibration curves were generated using Prism Graphpad. Sigmoidal 4PL was used to obtain the total aSyn curve and sigmoidal 5PL was used to obtain the pS129 aSyn curve. LLOD = mean blank + 3 times standard deviation of blank, LLOQ = mean blank + 10 times standard deviation of blank.
